# An Emerging Duck Egg-Reducing Syndrome Caused by a Novel Picornavirus Containing Seven Putative 2A Peptides

**DOI:** 10.3390/v14050932

**Published:** 2022-04-29

**Authors:** Xin Su, Dun Shuo, Yanqiu Luo, Xue Pan, Dawei Yan, Xuesong Li, Weishan Lin, Dongming Huang, Jianmei Yang, Chunxiu Yuan, Qinfang Liu, Qiaoyang Teng, Zejun Li

**Affiliations:** Department of Avian Infectious Diseases, Shanghai Veterinary Research Institute, Chinese Academy of Agricultural Sciences, Shanghai 200241, China; suxin900220@163.com (X.S.); dun.shuo@hotmail.com (D.S.); lyqyanqiu@foxmail.com (Y.L.); xue.pan@wur.nl (X.P.); yandawei865@163.com (D.Y.); lixuesong@shvri.ac.cn (X.L.); weishan09@126.com (W.L.); huajgdongming0402@163.com (D.H.); yangjianmei@shvri.ac.cn (J.Y.); yuanchx@shvri.ac.cn (C.Y.); liuqinfang@shvri.ac.cn (Q.L.)

**Keywords:** emerging, duck egg-reduction syndrome, picornavirus, 2A peptides

## Abstract

Since 2016, frequent outbreaks of egg-reducing syndromes caused by an unknown virus in duck farms have resulted in huge economic losses in China. The causative virus was isolated and identified as a novel species in Avihepatovirus of the picornavirus family according to the current guidelines of the International Committee on Taxonomy of Viruses (ICVT), and was named the duck egg-reducing syndrome virus (DERSV). The DERSV was most closely related to wild duck avihepatovirus-like virus (WDALV) with 64.0%, 76.8%, 77.5%, and 70.7% of amino acid identities of P1, 2C, 3C, and 3D proteins, respectively. The DERSV had a typical picornavirus-like genomic structure, but with the longest 2A region in the reported picornaviruses so far. Importantly, the clinical symptoms were successfully observed by artificially infecting ducks with DERSV, even in the contact exposed ducks, which suggested that DERSV transmitted among ducks by direct contact. The antibody levels of DERSV were correlated with the emergence of the egg-reducing syndromes in ducks in field. These results indicate that DERSV is a novel emerging picornavirus causing egg-reducing syndrome in ducks.

## 1. Importance

Duck egg-reducing syndromes caused huge economic losses to the poultry industry in China recently, however, the causative agent had kept unknown since the disease broke out. In this study, the virus was isolated, identified, and named the duck egg-reducing syndrome virus, which was a novel virus species belonging to *avihepatovirus* in the picornavirus family. Interestingly, the virus possesses the longest 2A region in all reported picornaviruses and might produce seven putative polypeptides. Our findings enriched the knowledge of picornaviruses and provided the key information for prevention and control of duck egg-reducing syndromes.

## 2. Introduction

Picornaviruses are non-enveloped viruses with positive-sense, single-stranded RNA genomes [1,2] ranging in size from 6.7 to 10.1 kilobases (kb), which typically contain a single open reading frame (ORF) flanked by the highly structured 5′ and 3′ untranslated regions (UTRs). Most of the Picornaviruses encode a large polyprotein except for the members of the species in genus *Dicipivirus* that have two internal ribosome entry sites (IRES) and encode two ORFs [1,3]. Picornaviruses are ubiquitous and globally distributed, and the number of newly discovered picornaviruses has increased dramatically in the past decade [4]. The family *Picornaviridae* was composed of 80 species and was divided into 35 genera in 2017 [5]. Up to now, the family of *Picornaviridae* consists of 158 species grouped into 68 genera (https://www.picornaviridae.com/index.html, accessed on 8 March 2021).

Although only a few viruses in the *Picornaviridae* family cause infectious diseases in humans or animals, huge economic losses were caused in the national health care system [4] and animal husbandry each year. In duck farms, duck hepatitis A virus (DHAV) in genus *Avihepatovirus* causes a highly fatal infectious disease in ducks under 6 weeks old [6]. In addition, Avian sapelovirus (ASV) in genus *Sapelovirus* causes growth retard in ducklings. Duck megrivirus (DMV) in genus *Megrivirus*, aalivirus A GL/12 (AalV-A) in genus *Aalivirus*, and wild duck avihepatovirus-like virus (WDALV) in genus *Avihepatovirus* were detectable by the reverse transcription-polymerase chain reaction (RT-PCR) in ducks, but the failure of isolation and proliferation of those viruses kept us from understanding their pathogenicity in ducks [7,8,9]. Since 2016, outbreaks of duck infectious disease characterized as egg production reduction caused by unknown pathogens frequently occurred in duck farms in China. The investigation of duck farms with declining egg production in many different areas, we found that the positive rate of DERSV is very high. According to our study, the epidemic firstly occurred in Anhui, China, but quickly spread to many other provinces in China, including Zhejiang, Jiangsu, Guangdong, and Shandong. The disease was characterized by a slow decline in egg production from the peak of 90% of laying rates to 50% of that in ducks. The decline in duck egg production has brought huge economic losses to duck farms. Importantly, the disease could not be cured by multiple antibiotics treatments; the known common avian viruses such as Tembusu virus, Adenovirus, Avian influenza viruses, Newcastle disease virus, Duck reovirus, Muscovy duck reovirus, Duck hepatitis virus 1, Duck hepatitis virus 3, Infectious bursal disease virus, Avian leukosis virus, Duck circovirus, Duck virusenteritis, Duck astrovirus, Goose parvovirus and Muscovy duck parvovirus were ruled out by PCR or RT-PCR in the diseased duck samples. Finally, a novel causative virus belonging to genus *Avihepatovirus* in the family of *Picornaviridae* was isolated and identified, and its genetic characteristics and pathogenicity in ducks were reported in this study.

## 3. Materials and Methods

### 3.1. Virus Isolation

In a duck farm in Anhui (AH) provinces, China, an outbreak of a disease is characterized by a slow decline in egg production from the peak of 90% of laying rates to 50% of that in ducks and by a long recovery period. The tissues of follicles, kidneys, and spleen samples of diseased ducks were collected and homogenized with phosphate-buffered saline (PBS). The polymerase chain reaction (PCR) was used to identify the virus using the primers for diagnosis of different known avian viruses including Tembusu virus and adenovirus that cause egg-loss symptoms in ducks, avian influenza viruses, Newcastle disease virus, duck reovirus, muscovy duck reovirus, duck hepatitis virus 1, duck hepatitis virus 3, infectious bursal disease virus, avian leukosis virus, Duck Circovirus, duck virusenteritis, duck astrovirus, goose parvovirus, muscovy duck parvovirus. The supernatant was filtered with a 0.22 μm filter and inoculated into the allantoic cavity of 14-day-old goose embryonated eggs for virus isolation (100 μL per egg). The goose embryonated eggs were incubated in a humidified egg incubator at 37 °C, and their survival was checked every day. The harvested allantoic fluid or/and embryos of the eggs that died 24–144 h post-inoculation (hpi) was collected. Virus-infected goose embryo allantoic fluid was purified by the limited dilution method three times. After purification by the limited dilution method, we identified the known avian viruses by PCR and RT-PCR again. Ten-fold serial dilutions of the virus-infected embryo allantoic fluid (0.1 mL) were inoculated into the allantoic sacs of 9-day-old SPF embryonated duck eggs and incubated for 144 h at 37 °C. Three eggs were used for each dilution. The embryo allantoic fluid of duck eggs were collected at 144 h post-inoculation and detected by RT-PCR; the 50% egg infectious dose was calculated by using Reed and Muench’s method. The embryo allantoic fluid had an infectivity titer of 10^6.5^ EID50/mL.

### 3.2. Observation under Electron Microscopy

The virus was enriched by ultracentrifugation and purified by 20%, 40% and 60% (*w/v*) discontinuous sucrose density gradient and centrifuged at 280,000× *g* (Break 0) for 5 h. The purified virus was stained with phosphotungstate acid, and observed under the transmission electron microscope (TEM, H-7500, Hitachi, Japan).

### 3.3. Nucleic Acid Extraction and Identification

Nucleic acid was extracted from purified virus by using the TIANamp virus RNA Kit (Tiangen Biotech, Co., Ltd., Beijing, China) or the Viral DNA Extraction Kit (Sangon Biotech, Co., Ltd., Shanghai, China), according to the manufacturer’s instructions. To determine the nucleic acid type, extracted nucleic acid was treated with RNase and DNase (Takara Biotechnology, Dalian, China), according to the manufacturer’s recommendations.

### 3.4. Construction of a CDNA Library of Viral Genes

Viral nucleic acid library was constructed by sequence-independent amplification using the viral RNA extracted by TIANamp virus RNA Kit. The first-strand cDNAs were synthesized with random reverse transcription primer of K-8N (GACCATCTAGCGACCTCCACNNNNNNNN) according to the previous description [10]. On the opposite strand of the cDNA, single round priming and extension was performed using Klenow fragment (TaKaRa Biotechnology, Dalian, China).

PCR of extension products was performed using 5 uL of the reaction described above in a total reaction volume of 50 uL containing 5U TaKaRa Ex Taq, 100 mM 10× Ex Taq Buffer (Mg2 + plus), 10 mM dNTPs (TaKaRa Biotechnology, Dalian, China), and 1 mM primer K (GAC CAT CTA GCG ACC TCC AC). Products were identified by agarose gel electrophoresis and the fragments larger than 250 bp were isolated and subcloned into pMD-19T vector for sequencing [10,11].

### 3.5. Nucleotide Sequencing and Phylogenetic Analysis

Plasmids extracted from the bacterial clones picked randomly from the cDNA library were directly sequenced. To compile and analyze the sequences, we used the SEQMAN program (DNASTAR, Madison, WI, USA). Putative ORF and deduced amino acid sequences of the viral genome was predicted using ORF finder (https://www.ncbi.nlm.nih.gov/orffinder/, accessed on 11 May 2018). The sequences of the reference viruses were obtained from GenBank by using the basic local alignment search tool (BLAST) at the National Center for Biotechnology Information (NCBI) (http://blast.ncbi.nlm.nih.gov/Blast.cgi, accessed on 14 May 2018).The secondary structure of IRES was predicted by RNAstructure (http://rna.urmc.rochester.edu/RNAstructureWeb/Servers/Predict1/Predict1.html, accessed on 15 May 2018), and drawn with StructureEditor. The conserved domains in the structural and nonstructural proteins were predicted using the conserved domain database (CDD) search [12]. The nucleotide sequences were compared with the sequences of reference viruses (Table A1) with the MEGALIGN program (DNASTAR) by using the Clustal alignment algorithm [6,13]. The phylogenetic trees were generated with 68 genera from the family of Picornaviridae by MEGA software (MEGA version 7.0). The possible cleavage sites of the polyprotein of DERSV were mapped based on (i) NetPicoRNA predictions [14], and (ii) the amino acid alignment with the selected strains of the closest relatives WDALV, AalV-A, DHAV, and (iii) the preference of picornavirus 3Cpro for the small amino acid residue (e.g., Q, E, G, S, R, M, A, and N) at the P1 position [7,14].

### 3.6. Study of the Correlation of DERSV between DERSV Infection

To understand the relationship between DERSV and duck egg production decline, diseased duck samples with egg decline were collected in different duck farms distributed in AH, Shandong (SD), Jiangsu (JS), and Guangdong (GD), and DERSV was detected. Some of the DERSV positive samples were selected according to the areas and inoculated with 9-day-old duck eggs for virus isolation, whole-genome sequencing, and further analysis.

Serological surveillance was conducted in 5 h in a breeding duck farm from October to December 2020. The average egg production rate was recorded every day, and each of 20 serum samples in each duck house were collected randomly at three-time points. The specific DERSV antibodies in the sera were detected by a blocking enzyme-linked immunosorbent assay. Briefly, the purified DERSV was diluted to 0.11 μg/well with 0.1 M bicarbonate buffer (Ph 9.6) and coated on the enzyme-linked immunosorbent assay (ELISA) plate. After incubating at 4 °C overnight, it was washed with PBS (pH 7.4) containing 0.05% Tween-20 (PBST) for 3 times. The blocking buffer (phosphate buffer containing 5% skim milk, 100 μL/well) was added to the reaction plate and sealed at 37 °C for 1 h. Serum samples were diluted 10 times with PBS and incubated on the ELISA plate for 1 h at 37 °C. The wells were then washed 3 times with PBST and incubated with monoclonal antibody (MAb)3G11(1:10) for 1 h at 37 °C. The MAb 3G11 can specifically react with DERSV but does not cross-react with other potentially infected duck viruses such as avian influenza viruses, Newcastle disease virus, duck hepatitis virus 1, duck hepatitis virus 3, goose parvovirus, duck Tembusu virus, adenovirus, duck reovirus. After washing the plate 3 times, goat anti-mouse IgG (1:2000, Sigma, St. Louis, MO, USA) conjugated with horseradish peroxidase (HRP) was added and incubated for 1 h at 37 °C. After rinsing with PBST for 3 times, add 3,3′,5,5′-tetramethylbenzidine 100 μL and incubate at room temperature for 10 min. Then, 0.1N of sulfuric acid was added to stop the reaction. Measure the optical density (OD) of the wells at 450 nm, and the percent inhibition (PI) value was calculated according to the formula: PI (%) = [1 − (OD450 sample/OD450 negative-control sample)] × 100. The serum was considered positive for DERSV reactivity when the PI value was ≥22.1%.

### 3.7. Animal Study

To determine the pathogenicity and transmissibility of DERSV, eight laying shelducks were inoculated intramuscularly with 10^5.5^ EID50 of DERSV in a volume of 200 μL. One day later, eight naive laying shelducks were introduced into the same isolator to test the transmissibility of DERSV. The inoculated ducks were euthanized on 6 dpi, and the exposed ducks were euthanized one day later, and the liver, kidney, and follicles samples were collected. The serum samples were collected from the remaining inoculated ducks at 2, 4, and 6 dpi and from the contact ducks at 2, 4, and 6 dpc for antibody detection. All samples were collected for virus isolation by using 9-day-old embryonated duck eggs and detected by RT-PCR. Additionally, we identified the known avian viruses by PCR and RT-PCR again. For histopathological analyses, samples were fixed with 4% paraformaldehyde, sectioned, and pathological observation of tissue sections after staining with hematoxylin and eosin. Alternatively, immunohistochemical analysis was conducted with a Mab(3G11) against the DERSV.

## 4. Results

### 4.1. Isolation of a Novel Virus

Since 2016, outbreaks of a duck egg-reduction syndrome caused by unknown pathogens were reported in AH, SD, JS, and GD provinces in China. Various antibiotics were used to mitigate the outbreak in ducks without any success, suggesting that the pathogen of the duck egg-reduction syndrome might be a viral pathogen. To identify the pathogen, the known avian viruses such as Tembusu virus and Adenovirus that cause egg drop symptoms in ducks, Avian influenza viruses, Newcastle disease virus, Duck reovirus, Muscovy duck reovirus, Duck hepatitis virus 1, Duck hepatitis virus 3, Infectious bursal disease virus, Avian leukosis virus, Duck circovirus, Duck virusenteritis, Duck astrovirus, Goose parvovirus, Muscovy duck parvovirus were detected by PCR or RT-PCR in the diseased duck samples, and all results were negative, which suggested that the pathogen might be a novel virus. To isolate this virus, a homogenate of the follicles, kidneys, and spleen samples collected from ducks with the egg-reduction syndrome were inoculated into the allantoic cavity of 14-day-old goose embryonated eggs. All the infected goose embryonated eggs died after 120 hpi. The allantoic fluid of the dead goose embryonated eggs was harvested. After three times of limited dilution purification in goose embryonated eggs, the purified virus was harvested and detected for the above-mentioned known viruses, which were all ruled out in the isolated virus. Therefore, the virus of the egg-reduction syndrome may be a novel unknown virus.

### 4.2. A Novel RNA Virus Was Identified and Named as DERSV

In order to further characterize the novel virus, the virus was purified by sucrose density gradient centrifugation and observed under a transmission electron microscope. The virion was spherical and unenveloped with a diameter of approximately 25–30 nm. Moreover, the nucleic acids extracted from the purified virus were resistant to DNase but not to RNase, which suggested that this virus is an RNA virus.

To determine the genome sequence of this RNA virus, the cDNA libraries were constructed by sequence-independent amplification using primer K-8N. A total of 100 positive clones were sequenced, and a continuous sequence (about 2700 bp) was obtained. The remaining sequence of the viral genome were amplified segmentally by RT-PCR using the primer walking process and by rapid amplification of the cDNA terminal [14,15]. The whole genome sequence was confirmed by seven overlapping DNA fragments amplified by RT-PCR with specific primers. Finally, the complete genome sequence of this virus was successfully obtained, which was in a length of 9026 bp (GenBank accession no. OL956952).

**Figure 1:**
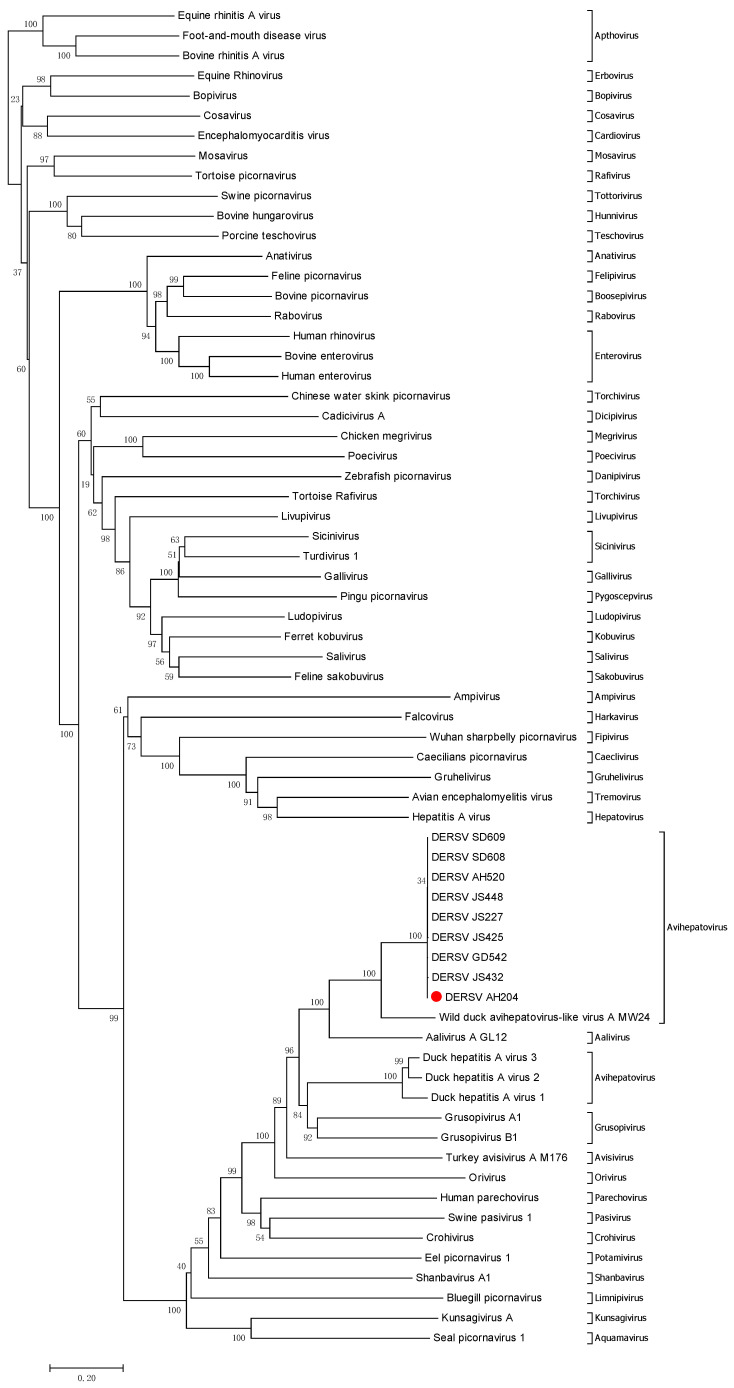
Phylogenetic relationship between DERSV and other picornaviruses. The neighbor-joining tree was constructed according to the amino acid sequences of DERSV (AH204 strain). The numbers along the branches mark the bootstrap values percentage out of 1000 bootstrap resamplings. The scale bar indicates the number of substitutions per site.

Using BLASTx searches, this new virus shared 45.2–66.6% nucleotide homology with that of the picornaviruses, which suggested that this virus was a kind of new picornavirus. Then, a phylogenetic tree was drawn to analyze the relationship between the new virus and the known picornaviruses including newly discovered picornavirus genuses such as *Ailurivirus, Anativirus, Boosepivirus, Bopivirus, Caecilivirus, Tropivirus,* etc. in recent years (Table A1). The result demonstrated that the virus was close to WDALV, AalV-A, and duck hepatitis A virus 1/2/3 that were picornaviruses from ducks (Figure 1). According to the new 2020 ICTV taxonomy (https://ictv.global/taxonomy, accessed on 8 March 2021), different members of a genus mean significant divergence (number of differences per site between sequences) of the orthologous proteins exceed 66% of P1 and 64% of 2C, 3C, and 3D. To further define the classification of the new virus, we analyzed the amino acid homology of P1, 2C, 3C, 3D between DERSV and DERSV-related picornavirus, such as WDALV, AalV-A and duck hepatitis A virus 1/2/3.Detailed analysis revealed that the new virus shared higher amino acid identities with WDALV (64%, 76.8%, 77.5%, and 70.7% in P1, 2C, 3C, and 3D proteins), AalV-A (35.6%, 62.2%, 59.3%, and 57.3% in P1, 2C, 3C, and 3D proteins), and duck hepatitis A virus 1 (35.1%, 52.1%, 45.0% and 47.6% in P1, 2C, 3C, and 3D proteins) (Table 1). Thus, the new virus in this study belongs to genus *Avihepatovirus* as well as duck hepatitis A virus described as previously [16,17]. However, this novel virus is different from WDALV, AalV-A and duck hepatitis A virus based on the large divergence in the amino acid and nucleotide sequence levels. Given this novel virus mainly causes egg drop in the ducks, this virus was named as duck egg-reducing syndrome virus (DERSV).

### 4.3. DERSV Had a Typical Picornavirus-Like Genomic Structure

**Figure 2:**
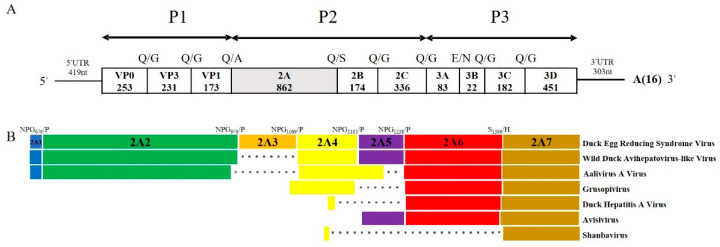
Schematic diagram of whole-genome and 2A region. (**A**) Predicted genome organization of the DERSV. P1 represents viral structural proteins, and P2 and P3 represent non-structural proteins. The length of amino acid was indicated in each gene box. (**B**) Comparison of putative 2A peptides of Picornaviruses. The dot represents the absence of amino acids and the different colors in the box represent 2A1(blue), 2A2(green), 2A3(orange), 2A4(yellow), 2A5(purple), 2A6(red), 2A7(golden).

The complete polyadenylated RNA of the DERSV comprised 9026 nt excluding the poly(A) tail and had a single 8394 nt ORF flanked by 5′UTR (419 nt) and 3′UTR (303 nt), encoding a putative polyprotein precursor of 2768 amino acids (Figure 2A).

The virus had the typical picornavirus-like genome organization. The possible cleavage sites of the polyprotein of DERSV were mapped based on (i) the amino acid alignment with the selected strains of the closest relatives WDALV, AalV-A, DHAV, and (ii) NetPicoRNA predictions [14]. The Q/G (Gln/Gly), E/G (Glu/Gly), and Q/S (Gln/Ser) cleavage sites of 3Cpro were strongly supported by the polyprotein alignments and the NetPicoRNA predictions [6]. There was no evidence of the presence of the potential L-protein and the cleavage of VP0 into VP4 and VP2. Thus, the DERSV polyprotein might consist of capsid proteins P1 (253aa-VP0, 231aa-VP3, 173aa-VP1), and nonstructural proteins P2 (862aa-2A, 174aa-2B 336aa-2C) and P3 (83aa-3A, 22aa-3B 182aa-3C, 451aa-3D) (Figure 2A).

Distinguishingly, the 2A region of the DERSV had six putative cleavage sites suggested that the virus might produce up to seven 2A peptides (Figure 2B). Five canonical cleavage sites of DxExNPG|P at amino acid sites of 676, 976, 1069, 1165, and 1238 of precursor polyprotein might result in five putative 2A peptides, a 2A1 (19 aa), a 2A2 (300 aa), a 2A3 (93 aa), a 2A4 (96) aa and a 2A5 (73aa) and a 281 aa peptide which might be cleaved at S1396|H to generate an additional two putative 2A products, 2A6 (158 aa) and 2A7 (123 aa). The S/H cleavage sites were not common among picornavirus, but are found in some picornaviruses, e.g., WDALV, DHAV-1 [16].

The 2A1 of DERSV shared 78.9% of amino acid homology with WDALV and 50% of amino acid homology with Foot-and-mouth disease virus. In contrast, the 2A3 is a unique peptide for the DERSV compared with other viruses in the *Picornaviridae*. Due to the insertion of 2A3, the 2A region of the DERSV is the longest in all the reported picornaviruses. The 2A2, 2A4, 2A5, 2A6, and 2A7 of DERSV possessed 44%, 69.6%, 58.9%, 51.9%, and 60.2% amino acid homology with that of WDALV. In addition, another predictive autocleavage motifs, GxExNPG788P, was found on 2A2, which suggested that more 2A peptides might be available in DERSV. Together with three characteristic sequence motifs (G1-G3) and GTP binding site of Ras-like-GTPase superfamily of small guanosine triphosphatases, the conserved motif of GxxGxGKS (x = any residue) potentially responsible for a 2C-like NTPase function was found in 2A6 (Table 2) [6]. Based on sequence alignment, the 2A6 was related to P-loop-containing nucleoside triphosphate hydrolases, and the 2A7 of DERSV was a parechovirus-like 2A with an H-box/NC-motifs (Table 2) [18].

**Figure 3:**
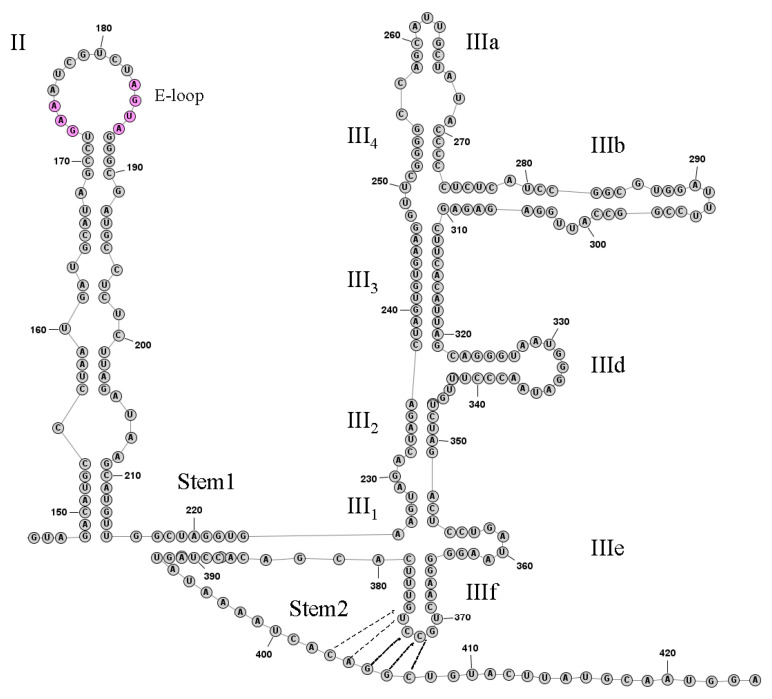
Predicted secondary structure of the DERSV IRES. Domains II and III are labeled according to corresponding domains in the type IV IRESs [14]. In domain III, individual helical segments are labeled III1 and III2, etc.; and individual hairpins are labeled IIIa and IIIb, etc. The pseudoknot stem 1, stem 2 and domain IIIf helix are labeled.

The predicted 419 nt 5′UTR of DERSV was significantly shorter than the most of picornaviruses. The secondary structure of the 3’-proximal portion of the DERSV 5′UTR was predicted by using RNA structure (http://rna.urmc.rochester.edu/RNAstructureWeb, accessed on 15 May 2018), which revealed that the region from nt145 to nt424 showed structural similarity to hepacivirus/pestivirus (HP)-like type IV IRES with stem-loop domains II and III and the predicted initiation codon was mapped at position 417 that was surrounded by the optimal Kozak context (GxxAUGG) (Figure 3) [19,20,21].The DERSV Domain II consisted of 67 nucleotides that contained the E-loop GAA173–175 and AGUA183–186 motifs present in most type IV IRES elements. Domain III in DERSV included a series of signature elements characteristic of HP-like IRES in domain IIId and domain IIIe. Interestingly, the DERSV domain IIIe consisted of a 3 nt stem (5′-CCU/5′-GGA) and five unpaired highly conserved GAUA motifs that were distinct from aalivirus (5′-CUC/5′-GAG) and all other those of other HP-like IRESs(5′-GGGU/5′-UCGGG) reported to date. Similar to classical swine fever virus, feline picornavirus 1, and bat picornaviruses 1 and 2 [19,22,23,24], the subdomain IIId of DERSV IRES contained an unpaired GGG motif within the terminal loop.

Analysis of the capsid regions revealed that DERSV contained a potential myristylation sequence (GxxxxS/T) at positions 155–159. This location is not quite similar to those of AalV-A at positions 39–43, DHAV at positions 31–35 [16], canine picornavirus 1 at positions 50–54 [25] and feline picornavirus 1 at positions 51–55 [23]. Using the CDD search, two RHV-like domains were found at residues 64–252 and 346–480 in P1 polypeptides [12]. Similar to other picornaviruses, the nonstructural proteins of DERSV contained some conserved characteristic motifs including two characteristic motifs for helicase (GATGSGKS1251-1258) in 2A6 and 2C for nucleoside triphosphate (NTP) binding, (QEIHIFDDLGQ1873-1883) in 2C protein for putative helicase activity [26,27], a cysteine protease motif (GLCG2277-2280) in 3C protein [26], and RNA-dependent RNA polymerase(RdRp) motifs (KDELR2466-2470, GGMCSGSPCTTVLNT2593-2607, YGDD2633-2636 and FLKR2681-2684) in 3D protein.

### 4.4. DERSV Infection Is Correlated with the Decline of Egg Production in Ducks

To understand the relationship between DERSV and duck egg production decline in field, a total of 379 samples were collected from diseased ducks with egg decline syndromes in different duck farms distributed in AH, SD, JS, and GD for DERSV detection. The positivity rate of DERSV was 61.7% (234/379) detected by RT-PCR from diseased ducks’ tissues including, livers, spleens, pancreases, ovaries, intestines, oviducts, kidneys, which suggested that this virus has been widely spreading in the main duck-raising areas of China. The positive ratio of the virus was highest in tissues kidneys (75%, 69/92), ovaries (69.6%, 39/56). Then, the positive rate of the virus was also higher in livers (56.0%, 51/91), intestines (64.7%, 22/34), oviducts (55.9%, 19/34) spleens (50%, 24/48), pancreases (41.7%, 10/24). All these results show that DERSV is closely related to the decline of egg production in ducks. Eight tissue samples were selected from 234 of DERSV positive tissues from different regions and different times for virus isolation and sequencing, and compared with the early DERSV isolates. Sequence comparison showed that the nucleotide homology and amino acid homology between these DERSV isolates (GenBank accession no. OM259401, OM259402, OM259403, OM259404, OM259405, OM259406, OM259407, OM259408) were 99.9–100% and 99.7–100%, respectively, indicating that although the variation of duck egg reduction syndrome virus isolate was small, it was distributed in different duck breeds in many provinces.

### 4.5. The Close Relationship between DERSV Antibody Level and Egg Decline Syndromes in the Clinic

**Figure 4:**
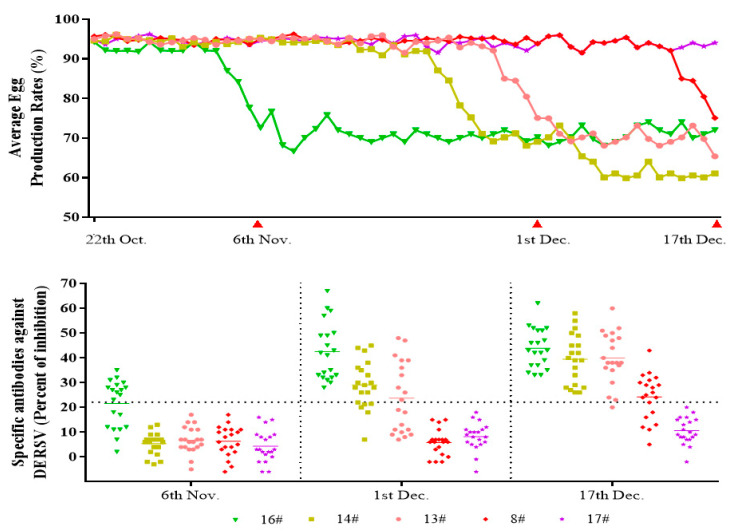
The correlation survey between the outbreaks of egg-reducing syndromes and the appearance of the specific antibodies against DERSV. In the early stage, the egg-reducing syndromes happened in one duck house. Meanwhile, the antibodies against DERSV were detectable in some serum samples, and seroconversion was found later in all the serum samples. In the other three duck houses, the egg-reducing syndromes happened one by one, and the antibodies against DERSV changed in a similar dynamic pattern as in the first duck house. The DERSV antibodies were not detected in the house without the egg-reducing syndrome.

To further confirm whether DERSV caused the clinical symptoms of duck egg-reducing syndromes, the average egg production rate and the serological surveillance in 5 h in a breeding duck farm during the outbreaks of egg-reducing syndromes were monitored from October to December 2020. In the early stage, the egg-reducing syndromes were found only in house #16, whereas the egg production rates kept on a high level in the other four duck houses. The antibody against DERSV was detectable in a part of serum samples from ducks raised in the house where egg drop happened, whereas no positive samples were found in the other four duck houses. Twenty-five days later, the serum samples were collected when the egg-reducing syndromes happened in houses #13, #14. The antibody against DERSV turned positive in some serum samples collected in those two duck houses. At the same time, all of the serum samples collected in the house occurred the egg drop disease firstly convert positive to DERSV antibody, whereas the serum samples collected in the remaining two houses with normal egg production showed negative to DERSV antibody. Sixteen days later, the serum samples were collected when the egg drop disease happened in another duck house (8#). The DERSV antibody showed a similar covert pattern in the new outbreak house as in those three houses broke out early. At that time, the egg production rates in those three early outbreak houses kept at a lower level and showed no signs of recovery (Figure 4). The emergence of the egg reducing syndrome and the development of DERSV antibodies were correlated in this farm, suggesting that DERSV was a causative agent of the duck egg-reducing syndrome.

### 4.6. Pathogenicity of DERSV in Ducks

**Figure 5:**
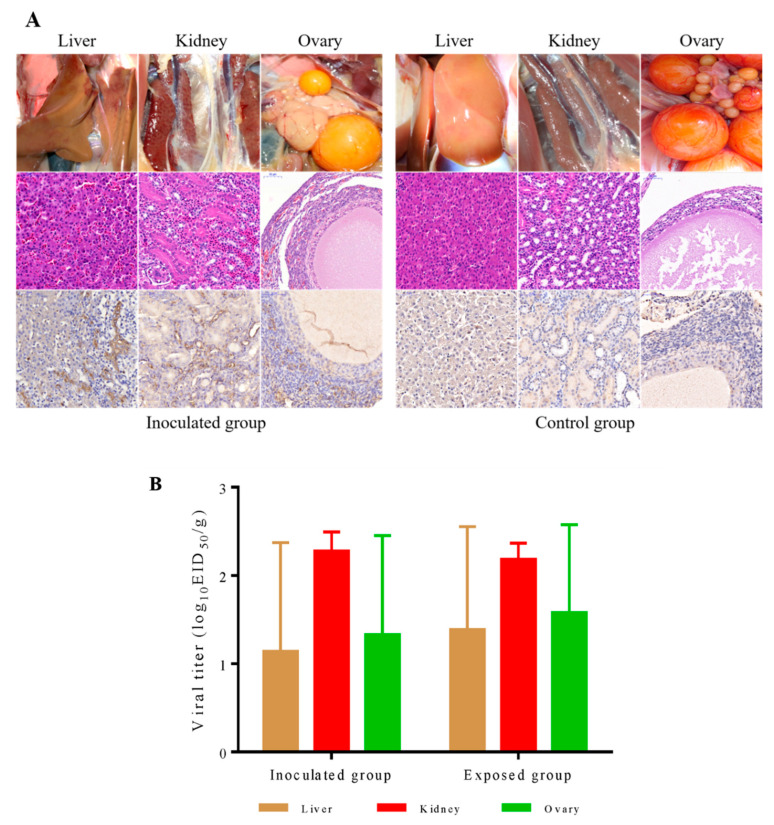
Pathogenicity of DERSV ducks. (**A**) Infected ducks show liver and renal hemorrhage, follicular hemorrhage, and rupture. Congestion of hepatic venules and sinusoid, congestion of renal interstitium, degeneration of renal tubular epithelial cells, and Necrosis of follicular epithelial cells were detectable on the tissue sections accordingly. In addition, viral antigens could be observed in those tissues of infected ducks. (**B**) Virus titer in tissues of livers, kidneys, and ovaries of inoculated and exposed ducks was determined on 9-day-old embryonated duck eggs. The lower limit of virus detection was 0.5log10 EID50 per gram tissue.

To evaluate the pathogenicity of the DERSV in ducks, we infected eight laying shelducks by inoculating them intramuscularly (i.m.) with 10^5.5^ EID_50_ of DERSV in a volume of 200 μL. One day after inoculation, eight naive laying shelducks were introduced into the isolator where the i.m.-inoculated ducks were housed. The inoculated ducks or contact exposed ducks showed a certain percentage of tissue damage including significant follicular hemorrhage and rupture (3/8 inoculated ducks, 2/8 exposed ducks), liver hemorrhage (5/8 inoculated ducks, 4/8 exposed ducks), and kidney (7/8 inoculated ducks, 6/8 exposed ducks) (Figure 5A). After hematoxylin and eosin staining of impaired ovary, liver and kidney sections, the result demonstrated that necrosis of follicular epithelial cells(ovary), hyperemia of hepatic venules and sinuses(liver), hyperemia of the renal interstitium, and degeneration of renal tubular epithelial cells(kidney) (Figure 5A). In addition, viral antigens were detected in ovary, liver, and kidney sections with pathological changes.

To determine the distribution of the DERSV in ducks, we detected the virus in the ovary, liver, kidney tissues, and serum samples. In the inoculated ducks, DERSV replicated best in the kidneys with a titer of 10^2.28 ± 0.22^ EID_50_/g, followed by the ovaries (a titer of 10^1.33 ± 1.12^ EID_50_/g) and liver (a titer of 10^1.14 ± 1.23^ EID_50_/g) (Figure 5B). In the contact exposed ducks, the replication level of DERSV in kidney (a titer of 10^2.18 ± 0.19^ EID_50_/g), ovary (a titer of 10^1.58 ± 0.99^ EID_50_/g) and liver (a titer of 10^1.39 ± 1.17^ EID_50_/g) were similar to those in inoculated ducks (Figure 5B). Moreover, DERSV was first isolated (1/8) from blood on the 2 dpi, the infected group had a positive rate of 50% (4/8) on the 4 dpi and the highest rate of 75% (6/8) on the 6 dpi in the blood samples of inoculated ducks. In the contact exposed ducks, this virus started to be isolated on 4 dpc with a positive rate of 25% (2/8) and 62.5% (5/8) on 6 dpc (Table 3). All these results suggested that this virus was transmitted among ducks by contact. Finally, we identified the known virus by RT-PCR again. The known viruses could not be detected by PCR or RT-PCR again. These suggest that these clinical symptoms of ducks were caused by infection with DERSV and were not mixed with known viruses.

## 5. Discussion

A stunning increase in newly identified viruses in the past decade has shown that picornaviruses have an extremely wide range of hosts distributed globally [4,28]. Moreover, picornaviruses exhibit a surprising diversity of genome sequences and genome layouts, challenging the definition of taxonomic relevant criteria. In this study, we identified a novel virus species named as DERSV, which caused duck-egg-drop syndrome. The virus was most closely related to WDALV and AalV-A. Based on current guidelines defined by ICTV, the amino acid identities in P1, 2C, 3C, and 3D regions among members of a genus should ≥34%, ≥36%, ≥36%, and ≥36%, respectively. Based on the rules, DERSV could be classified into the genus *Avihepatovirus* or the genus *Aalivirus*. However, DERSV shared higher amino acid identities with WDALV, which belongs to the genus *Avihepatovirus*. As a result, we proposed that DERSV was a novel species in the genus *Avihepatovirus*. So far, both AalV-A and WDALV were failed to be isolated, although they were detected in ducks [8,29], and the pathogenicity of those viruses in ducks remains unknown for the lack of animal infection experiment.

The 2A protein is the least conserved non-structural protein in picornavirus, involved in the virus assembly and virulence [30,31,32,33]. AalV-A has six putative 2A peptides, which is the picornavirus with the largest number of 2A peptides reported so far [8,32]. Interestingly, DERSV possesses a total of up to seven putative 2A peptides. The first 6 2A peptides were separated by the “DxExNPGP” motif and might induce a co-translational “cleavage” event called ribosome “skipping,” to be separated at the “G/P” site. There are three possibilities for 2A-mediated ‘ribosome-skipping’ after the release of the nascent polypeptide: (1) jump successfully and continue to translate 2A downstream protein; (2) jump successfully, but ribosomes terminate translation; or occasionally, producing only 2A upstream protein; (3) jump failure, continue translation to produce fusion protein [34]. DxExNPGP motif induces the “cleavage” event of co-translation, enabling P1-2A to be released from P2 polyproteins [33]. There have been reported that the D mutation in the “DxExNPGP” motif of the foot-and-mouth disease virus lost this function, but some studies have exhibited that the key amino acids in this motif are NPGP and the “GxExNPGP” in DHAV-1 2A1 can still function successfully [33]. In DERSV 2A2, we found an additional GxExNPG788P motif, which might result in up to 8 2A peptides of DERSV. Interestingly, a unique peptide 2A3 of DERSV was found firstly in this study. 2A proteins or peptides can resist host immune response and promote virus replication [33]. Most of the avian picornaviruses contained multiple mature 2A peptides, suggesting that the mechanism by which picornaviruses infect avian hosts may be more complex than other picornaviruses [35]. The function of DERSV 2A3 in virus replication need to be explored further.

To date, five distinct types (I, II, III, IV, and V) of picornavirus IRES elements have been recognized. The picornavirus type IV IRES structures resemble HP IRESs in key respects, notably in comprising domains II and III, and containing a series of signature elements in domain III [7,19,36]. According to previous reports, it was found that picornavirus HP-like IRESs were divided into three groups A, B and C, and most of the picornavirus HP-like IRESs belonged to group B [6,7,19,22,37,38,39]. It is worth noting that the secondary structure model of the inferential domain III of DERSV S is highly conservative in Aalivirus, pigeon picornaviruses B, Quail picornavirus, Mesivirus, duck megrivirus [7,8,38,39].

The picornaviruses are important pathogens in humans and animals, causing diseases that affect the central nervous system, respiratory and gastrointestinal tract, heart, liver, pancreas, skin, and eyes [4]. Although many new picornaviruses were found in animals through high-throughput sequencing recently, little is known about the pathogenicity of most of those viruses in their natural hosts for lack of virus isolation methods [9,40]. So far, only two duck picornavirus species the DHAV and the ASV-1 were isolated from domestic duck, whereas three duck picornavirus DMV, AalV-A, and WDALV were detected but failed to isolate so far [8,29]. The virus reported in this study was a novel species belonging to the genus of the *Avihepatovirus* genus. However, the main clinical signs and symptoms of the disease caused by the virus were the duck egg-reducing and follicular hemorrhage and rupture. To distinguish with duck hepatitis virus, we suggest naming the novel virus as duck egg-reducing syndrome virus according to the main clinical signs caused by the virus in ducks.

Both DERSV and Duck egg drop syndrome virus (DEDSV) lead to a drop in egg production, but they are quite different: DERS is a disease caused by picornavirus infection. The main clinical symptoms are that the laying rate slowly decreases from a peak of 90% to 50%, and the laying rate will rise after a period of time, and infected ducks will not die. DEDS is a disease caused by DEDSV (flavivirus) infection, and its main clinical symptoms are anorexia, diarrhea, ataxia, paralysis and, most importantly, a significant decline in egg production, which rapidly dropped to less than 10% [41]. Some of the affected ducks showed neurological disorders characterized by progressive paralysis, with a mortality rate of between 5% and 30% [41].

At present, in the absence of specific DERSV treatment, vaccination will be the most feasible strategy to control DERSV. In view of the successful experience of picornavirus vaccines such as foot-and-mouth disease virus vaccine [42], poliovirus vaccine [43], and hepatitis virus vaccine [44], DERSV vaccine may become the main means to control the disease in future.

Taken together, our results indicated that a novel virus named was DERSV was isolated and identified from egg-reducing syndrome ducks. This virus belongs to the avian hepatitis virus family of picornavirus and has a typical picornavirus-like genomic structure. In the field, the antibody level of DERSV was closely related to the egg-reducing syndrome.

## Figures and Tables

**Table 1:** Comparisons of Amino acid sequence homology of DERSV(AH204) with the closely related picornaviruses.

Region	Wild Duck Avihepatovirus-Like Virus(Avihepatovirus)#	Duck Hepatitis A Virus 1 (Avihepatovirus)	Aalivirus A GL/12 (Aalivirus)	Grusopivirus A1(Grusopivirus)
P1	64.00%	35.10%	35.60%	28.70%
2C	76.80%	52.10%	62.20%	48.60%
3C	77.50%	45.00%	59.30%	45.30%
3D	70.70%	47.60%	57.30%	49.30%

Note: # Virus (Genera).

**Table 2:** The seven 2A peptide of DERSV.

2A Peptide	Length (aa)	Sequence Motif and Possible Function/Activity
2A1	19	DxExNPGP/Ribosomal skipping mechanism
2A2	300	DxExNPGP/Ribosomal skipping mechanism
2A3	93	DxExNPGP/Ribosomal skipping mechanism
2A4	96	DxExNPGP/Ribosomal skipping mechanism
2A5	73	DxExNPGP/Ribosomal skipping mechanism
2A6	158	GxxGxGKS, xTx, DxxG/Apoptosis of host cells
2A7	123	H-NC box/Viral replication and cell proliferation

**Table 3:** Virus isolation and detection from serum.

Test Time	Inoculated Group	Exposed Group
2dpi ^a^(dpc ^b^)	-^c^-------	--------
4dpi(dpc)	-+^d^+---++	+------+
6dpi(dpc)	+-++-+++	+++-++--

^a^ dpi, days post inoculation. ^b^ dpc, days post contact. ^c^-, no virus was isolated and detected. ^d^+, virus was isolated and detected.

## Appendix A

**Table A1:** The list of some representative viruses in the picornavirus family.

Picornavirus Genus	Viruses	GenBank Accession No.	Genome Size (nt)
Aalivirus	Aalivirus A GL/12	KJ000691	8959
Ailurivirus	Aimelvirus	MF327529	8029
Ampivirus	Ampivirus	KP770140	9246
Anativirus	Anativirus	AY563023	8289
Aphthovirus	Foot-and-mouth disease virus	AF308157	8134
	Bovine rhinitis A virus	JN936206	7250
	Equine rhinitis A virus	NC_003982	7734
Aquamavirus	Seal picornavirus	NC_009891	6693
Avihepatovirus	Wild duck avihepatovirus-like virus	MH453803	8742
	Duck hepatitis A virus 1	DQ864514	7689
	Duck hepatitis A virus 2	EF067924	7769
	Duck hepatitis A virus 3	EU352805	7791
Avisivirus	Turkey avisivirus A M176	KC465954	7532
	Avisivirus B1	KF979333	7310
Boosepivirus	Bovine picornavirus	LC006971	7570
Bopivirus	Bopivirus	KM589358	7018
Caecilivirus	Caecilians picornavirus	MG600103	8318
Cardiovirus	Encephalomyocarditis virus	MH191297	7734
Cosavirus	Cosavirus	FJ438903	7335
Crahelivirus	Crahelivirus	KY312540	7443
Crohivirus	Crohivirus	AB937989	7321
Danipivirus	Zebrafish picornavirus	MH368041	8298
Diresapivirus	Diresapivirus	KJ641685	6624
Dicipivirus	Cadicivirus A	JN819202	8755
	Bovine enterovirus	D00214	7414
Enterovirus	Human enterovirus	AY697458	7438
	Human rhinovirus	JX074057	6979
Erbovirus	Equine Rhinovirus	X96871	8828
Felipivirus	Feline picornavirus	JN572115	7391
Gallivirus	Gallivirus	JQ691613	8496
Gruhelivirus	Gruhelivirus	KY312541	7073
Grusopivirus	Grusopivirus A1	KY312544	7917
	Grusopivirus B1	KY312545	7104
Harkavirus	Falcovirus	KP230449	8003
Hemipivirus	Hemipivirus	MG600089	7939
Hepatovirus	Hepatitis A virus	NC_001489	7478
Hunnivirus	Bovine hungarovirus	JQ941880	7583
Kobuvirus	Ferret kobuvirus	KF006985	8052
Kunsagivirus	Kunsagivirus A	KC935379	7272
Limnipivirus	Bluegill picornavirus 1	NC_018506	8050
Livupivirus	Livupivirus	KX463670	7768
Ludopivirus	Ludopivirus	MF358731	8051
Marsupivirus	Burpengary virus	MK882499	6821
Malagasivirus	Lesavirus	KM396707	7687
Megrivirus	Chicken megrivirus	KF961186	9560
Mosavirus	Mosavirus	JF973687	6964
Mischivirus	Miniopterus schreibersii picornavirus	JQ814851	8468
Mupivirus	Mupivirus	KY432924	8422
Orivirus	Orivirus.	KM203656	7037
Myrropivirus	Myrropivirus	MG600081	7994
Oscivirus	Turdivirus	GU182408	7641
Parechovirus	Human parechovirus	L02971	7339
Parabovirus	Parabovirus	KY432927	8315
Pasivirus	Swine pasivirus 1	NC_018226	6916
Passerivirus	Turdivirus 1	NC_014411	8035
Pemapivirus	Pemapivirus	MG600106	9196
Potamipivirus	Eel picornavirus 1	KC843627	7496
Poecivirus	Poecivirus	KU977108	7653
Pygoscepivirus	Pingu picornavirus	MH255796	7601
Rabovirus	Rabovirus	KP233897	7853
Rafivirus	Tortoise Rafivirus	KJ415177	7204
Rajidapivirus	Rajidapivirus	MG600093	7947
Rosavirus	Rosavirus	JF973686	8749
Rohelivirus	Rohelivirus	KX156153	7780
Sapelovirus	Porcine sapelovirus	NC_003987	7491
Sakobuvirus	Feline sakobuvirus A	KF387721	7807
Salivirus	Salivirus	GQ179640	7982
Shanbavirus	Shanbavirus A1	KJ641698	7264
Senecavirus	Senecavirus	KR063107	7270
Sicinivirus	Sicinivirus	KF741227	9276
Symapivirus	Symapivirus	MG600076	8591
Teschovirus	Porcine teschovirus	LC386154	7138
Tottorivirus	Swine picornavirus 1	LC113907	7062
Torchivirus	Tortoise picornavirus	KM873611	7093
Tremovirus	Avian encephalomyelitis virus	NC_003990	7032
Tropivirus	Chinese water skink picornavirus	MG600091	8049
